# Renal Denervation in a Real Life Setting: A Gradual Decrease in Home Blood Pressure

**DOI:** 10.1371/journal.pone.0162251

**Published:** 2016-09-15

**Authors:** Martine M. A. Beeftink, Wilko Spiering, Michiel L. Bots, Willemien L. Verloop, Rosa L. De Jager, Margreet F. Sanders, Evert-jan Vonken, Peter J. Blankestijn, Michiel Voskuil

**Affiliations:** 1 Department of Cardiology, University Medical Center Utrecht, Utrecht, the Netherlands; 2 Department of Vascular Medicine, University Medical Center Utrecht, Utrecht, the Netherlands; 3 Julius Center for Health Sciences and Primary Care, University Medical Center Utrecht, Utrecht, the Netherlands; 4 Department of Nephrology & Hypertension, University Medical Center Utrecht, Utrecht, the Netherlands; 5 Department of Radiology, University Medical Center Utrecht, Utrecht, the Netherlands; Emory University Department of Medicine, UNITED STATES

## Abstract

**Objectives:**

To investigate the blood pressure dynamics after renal denervation through monthly home blood pressure measurements throughout the first 12 months.

**Methods:**

A cohort of 70 patients performed highly standardized monthly home blood pressure monitoring during the first year after denervation according to the European Society of Hypertension guidelines. At baseline and 12 months follow-up, office and ambulatory blood pressure as well as routine physical and laboratory assessment was performed.

**Results:**

Home blood pressure decreased with a rate of 0.53 mmHg/month (95% CI 0.20 to 0.86) systolic and 0.26 mmHg/month (95% CI 0.08 to 0.44) diastolic throughout 12 months of follow-up, while the use of antihypertensive medication remained stable (+0.03 daily defined doses/month, 95% CI -0.01 to 0.08). On average, a 12 month reduction of 8.1 mmHg (95% CI 4.2 to 12.0) was achieved in home systolic blood pressure, 9.3 mmHg (95% CI -14.2 to -4.4) as measured by 24-hour ambulatory blood pressure monitoring and 15.9 mmHg (95% CI -23.8 to -7.9) on office measurements.

**Conclusion:**

Blood pressure reduction after renal denervation occurs as a gradual decrease that extends to at least one-year follow-up. Home monitoring seems a suitable alternative for ambulatory blood pressure monitoring after renal denervation.

## Introduction

Hypertension is common in the western society and the risk of vascular complications is strongly related to blood pressure levels.[1] As the greatest contributor to cardiovascular morbidity and mortality hypertension is associated with 10.4 million premature deaths annually.[2] Despite a wealth of treatment options, blood pressure control is limited: only a third of patients receiving antihypertensive drugs are adequately controlled.[3]

In 2009, catheter-based renal denervation (RDN) was introduced as a new, promising treatment for patients with persistent hypertension despite comprehensive pharmacological treatment.[4] Initially RDN showed impressive results, mostly in cohort studies and some small randomized trials,[4–7] but more recent studies have shown mixed results for efficacy.[8–11] In the discussion following these results many gaps in the knowledge of RDN were identified, including issues concerning study design, patient selection, medication adherence as effect modifiers, the optimal procedural approach, anatomical variation and the lack of a reliable marker of procedural success.[12,13]

Among these issues is the uncertainty when to expect a response of RDN on blood pressure. It is unknown whether BP acutely decreases shortly after the intervention or more gradually over the course of several months. Therefore, we investigated home blood pressure measurements (HBPM) throughout the first year after RDN treatment to elucidate the dynamics of BP following RDN.

## Methods

### Study population

This study was conducted at the University Medical Centre Utrecht and is part of the Dutch National Renal Denervation Registry (NCT02482103) that was approved by the Medical Ethics Committee of the UMC Utrecht.[14] The registry contains screening, procedural and follow-up data of all patients treated with RDN in the Netherlands. The requirement to obtain informed consent for the registry was waived by the Medical Ethics Committee. All patients provided written informed consent for the original RDN study they participated in, or provided verbal informed consent if the RDN procedure was performed as routine medical care. The study was conducted in accordance with the Declaration of Helsinki[15] and the Dutch Medical Research Involving Human Subjects Act (WMO).

For the current analysis, we studied a cohort of consecutive patients that performed HBPM throughout the first year after RDN for resistant hypertension (an office systolic BP ≥160 mmHg and/or a 24-hour SBP ≥135 mmHg, despite the use of ≥3 antihypertensive drugs at maximally tolerated doses) or the inability to be adequately treated for hypertension due to recorded intolerance for antihypertensive drugs (non-resistant hypertension). Before intervention, all patients were subjected to a thorough screening procedure including 24-hour ambulatory blood pressure monitoring (ABPM), to exclude pseudo-resistant hypertension, significant white coat effect and secondary causes, as previously described.[16] This screening includes temporary cessation of all antihypertensive drugs, if deemed safe, to avoid interference with the investigations and to obtain unconfounded BP measurements. Immediately after these investigations, BP medication was restarted at once. Physicians were asked not to change the antihypertensive medication unless absolutely necessary.

The final decision for eligibility for RDN was made by a multidisciplinary team, consisting of a vascular medicine specialist (WS), a nephrologist (PB), an interventional cardiologist (MV) and an interventional radiologist (EJV). Major exclusion criteria included ineligible renal artery anatomy, an estimated glomerular filtration rate (eGFR) <30 mL/min/1.73m², severe co-morbidity and patient refusal.[17] The RDN procedure was performed via transfemoral approach according to the respective instructions for use of the device. The choice for the type of RDN catheter was left to the discretion of the interventionalist.

### Home blood pressure monitoring (HBPM)

To perform HBPM, patients received an automated WatchBP Home device (Microlife Inc., Widnau, Switzerland). Patients were instructed according to the European Society of Hypertension recommendations[18,19] to perform HBPM every month for a total of 12 months, starting one week after RDN. The HBPM routine was started one week after RDN and each following measurement week was scheduled to start on the same day of the next month. Additionally, HBPM was performed during the medication-free screening prior to RDN.

HBPM measurements were to be taken in a seated position after 5 minutes of rest, two times in the morning (6-9AM) and two times in the evening (6-9PM) during seven consecutive days. All measurements were automatically stored to the device and uploaded to a secure internet site (BP@home, MobiHealth B.V., the Netherlands). Patients were unable to add, delete or change any measurements on the device or on the BP@home server. In accordance to the guidelines[18,19] measurements taken on the first day of every week were discarded to avoid non-representative measurements due to anxiety with the technique and weeks with less than 12 readings were excluded for analysis to secure the quality of the measurements.

The BP measurements from each HBPM period of seven days were used to calculate the mean systolic (SBP) and diastolic (DBP) home BP for each month. Medication use for each period was recorded based on prescription history and detailed history taking. Prescribed dosages of antihypertensive drugs for each time point were converted into defined daily doses (DDD) using conversion factors provided by the World Health Organization (WHO) Drug Classification[20]. The cumulative daily intake of antihypertensive drugs was calculated for each patient using the sum of all DDD’s. No toxicological urine or blood analyses to confirm medication adherence was performed.

### Routine follow-up data

Office BP, laboratory results, medical history and physical examination were registered during screening and at six and 12 months follow-up. Ambulatory BP monitoring was performed during screening and 12 months follow-up. A subgroup of patients repeated the medication-free period at 12 months follow-up as part of a different study protocol.[21] All BP measurements were performed on Microlife WatchBP 03 devices (Microlife Inc., Windau, Switzerland) in accordance to the ESH guidelines.[22] Readings for ABPM were taken at least every 30 minutes during day and night using appropriate cuff sizes and repeated if more than 30% of measurements failed. Serum creatinine was used to estimate renal function (eGFR) using the CKD-EPI formula.[23]

### Statistical analysis

Results are presented as mean ± standard deviation or as absolute number with percentages, unless otherwise specified.

Multilevel linear mixed effect models were used for analysis of BP over time. This model has several advantages in modelling changes over time over a repeated measures ANOVA. In particular the model is not hampered by missing data and it provides estimation of effect size and precision.[24] Time in months was entered as a continuous variable, and random intercepts as well as random slopes were allowed in the model as appropriate guided by the -2 Log Likelihood statistic. Only post-RDN measurements were entered into the model, to avoid interference of an artificial increase in medication caused by the medication-free screening period. Pre-selected variables were added to the unconditional model (model I) as fixed effects, resulting in model II (age, gender, antihypertensive medication) and model III (age, gender, antihypertensive medication, body mass index (BMI), estimated glomerular filtration rate (eGFR), smoking status and baseline office BP) to correct for their possible influence on BP slope. Daily use of medication (DDD) was entered into the model as time-varying variable, while baseline variables were added as time-independent variables. The HBPM changes over time were subsequently modelled in pre-specified subgroups of risk factors and potentially confounding factors. For continuous variables, stratification was performed below or above the median for the study population. Interaction terms for each variable were added to the model to test for significant differences between subgroups.

For body mass index, eGFR, office BP and ABPM the change between baseline and 12 months follow-up was assessed by means of the paired samples T-test, or Wilcoxon signed rank test when appropriate.

Results were considered statistically significant if the 95% confidence interval (CI) did not include 0 or if the two-tailed probability value (p-value) did not exceed 0.05. All analysis was performed with SPSS statistical software version 22 (IBM SPSS Data Collection, Chicago, Illinois, USA).

## Results

Our registry included 118 patients who were treated with RDN between June 2010 (the start of RDN at our facility) and May 2014. As of May 2011, all patients (n = 90) consecutively received an automated device to perform HBPM. Sixteen patients refused HBPM or did not have access to internet, resulting in 74 patients of whom HBPM was available. Four patients only performed measurements during the screening procedure and were excluded from analysis.

**Fig 1:**
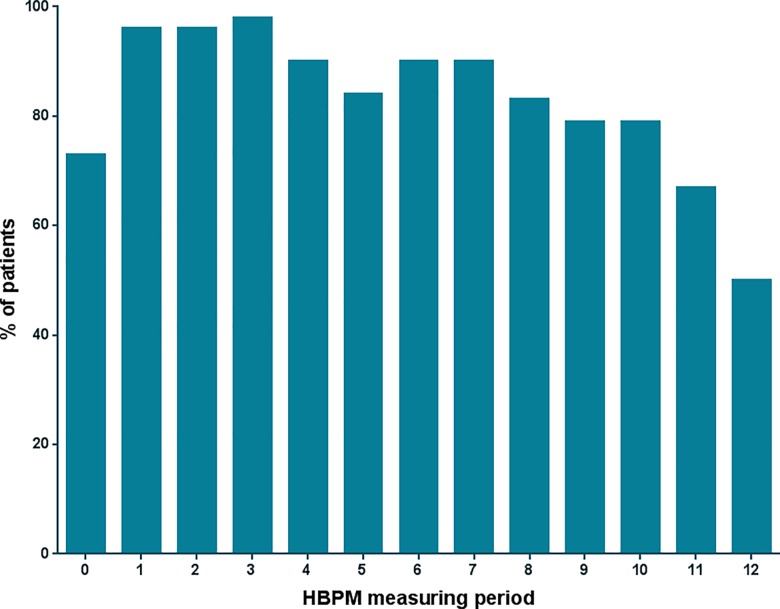
Amount of measurements contributing to HBPM. Figure depicts the percentage of patients completing home blood pressure measurements (HBPM) for each measuring period.

Characteristics of the remaining 70 patients are shown in Table 1. They performed a total of 756 HBPM periods after RDN (83% success rate). Five HBPM periods were discarded due to an insufficient number of measurements, all from a different subject. The average number of measuring periods was 11, with only three patients accomplishing less than six HBPM periods. The number of measuring periods contributing to each of the monthly averages is depicted in Fig 1. Fifty-two patients were treated with RDN for resistant hypertension, while eighteen patients were included with an inability to tolerate optimal pharmacological treatment due to documented intolerance to antihypertensive drugs. The mean amount of prescribed medication at baseline was 6.7 ± 3 for patients with resistant hypertension and 1.7 ± 1 for patients with intolerance to AHD.

**Table 1:** Patient characteristics.

Patient characteristics	N = 70
**Age,** years	59 ± 9
**Gender,** (male)	43 (63%)
**Caucasian**	67 (96%)
**Office SBP**, mmHg	191 ± 31
**Office DBP,** mmHg	104 ± 16
**BMI,** kg/m²	29 ± 5
**eGFR,** mL/min/1.73m²	80 ± 18
**CVD**	26 (37%)
**Dyslipidaemia**	35 (50%)
DM type 2*	22 (31%)
**No. of AHD,** median (min-max)	3 (0–6)
**Total DDD (sum of all AHD)**	5,4 ± 3,6
**Type of antihypertensive medication used**	
**diuretic**	54 (77%)
**RAAS inhibitor**	60 (86%)
**Aldosterone antagonist**	20 (29%)
**Beta-blocker**	42 (60%)
**Alpha-blocker**	12 (17%)
**Calcium-channel blocker**	46 (66%)
**Central acting antihypertensive**	2 (3%)
**Direct vasodilating drug**	3 (4%)
**No. of HBPM weeks,** mean (min-max)	11 (3–12)
**RDN device used**	
**Medtronic Symplicity**	66 (94%)
**St Jude Enlightn II**	3 (4%)
**Covidien OneShot**	1 (1%)
**Indication for RDN**	
**Resistant hypertension**	52 (74%)
**Non-resistant hypertension**	18 (26%)

Table legend: SBP systolic blood pressure, DBP diastolic blood pressure, BMI body mass index, eGFR estimated glomerular filtration rate, CVD cardiovascular disease, DM diabetes mellitus, AHD antihypertensive drugs, DDD daily defined dose, RAAS Renin-Angiotensin-Aldosterone System, HBPM home blood pressure monitoring, RDN renal denervation. RAAS inhibitors composed of angiotensin converting enzyme inhibitors, angiotensin receptor antagonists and direct renin inhibitors. Data is presented as absolute number (percentage) or as mean ± standard deviation, unless otherwise specified.

*DM type 1 was exclusion criterion for RDN treatment

Overall, the amount of antihypertensive medication prescribed decreased from 5.8±3.8 DDD at baseline to 5.3±3.6 DDD at 12 months with a mean difference of 0.5 (95% CI -1.3 to 0.2). There was no change in eGFR (Δ 0.33 ml/min/1.73m², 95% CI -2.1 to 2.8) or BMI (Δ 0.14 kg/m², 95% CI -0.33 to 0.6) between baseline and 12 months follow-up. In addition, there was no change in urinary sodium excretion (Δ 18 mmol/24hrs, 95% CI -34 to 70), although follow-up urine analysis was only available for 29 subjects.

### Home blood pressure measurements

Mean HBPM values decreased from 181/104 mmHg (SD 19/13) during the medication-free screening period before RDN to 158/92 mmHg (SD 17/13) one week after RDN and 152/87 mmHg (SD 21/13) at one year follow-up. Due to the medication-free baseline screening, the DDD differed from the baseline measurement to 1 week after renal denervation (0.5 vs 4.3), but remained stable from week 1 through month 12 (estimated slope 0.03 DDD/month, 95% CI -0.01 to 0.08).

The unconditional mixed model (model I) showed a significant change in BP of -0.56 mmHg/month after RDN (95% CI -0.89 to -0.24) for SBP and -0.27 mmHg/month for DBP (95% CI -0.46 to -0.07) during the 12 months after treatment (Table 2). In the full model adjusting for pre-specified variables (model III), BP change remained statistically significant at -0.53 mmHg/month (95%CI -0.86 to -0.20) for SBP and -0.26 mmHg/month (95% CI -0.44 to -0.08) for DBP.

**Table 2:** Change in home blood pressure over time after renal denervation.

	Estimate slope (mmHg/month)	95% CI
**Model I (unconditional)**		
Mean SBP	-0.56	-0.89;-0.24
Mean DBP	-0.27	-0.46;-0.07
Mean heart rate	-0.09	-0.18;-0.001
**Model II (adjusted for age, sex and AHD)**		
Mean SBP	-0.51	-0.84;-0.18
Mean DBP	-0.25	-0.44;-0.07
Mean heart rate	-0.09	-0.23;0.05
**Model III (full model)**		
Mean SBP	-0.53	-0.86;-0.20
Mean DBP	-0.26	-0.44;-0.08
Mean heart rate	-0.09	-0.23;0.04

SBP = systolic blood pressure, DBP = diastolic blood pressure, AHD = antihypertensive drugs (sum of all daily defined doses). The estimated slope represents the change in home blood pressure per month. Multivariable analysis in model III (full model) was adjusted for daily use of medication, age, sex, body mass index, estimated glomerular filtration rate, baseline office blood pressure and smoking

Heart rate changed significantly in the unconditional model, but this change was not clinically relevant (-0.09 bpm per month) nor statistically significant after correction for pre-specified variables. Results for the different subgroups are shown in Table 3.

**Table 3:** Change in home blood pressure over time after renal denervation in various strata of baseline patient characteristics.

	N =	estimate slope (mmHg/month)	95% CI	p-value for interaction
resistant hypertension	52	-0.72	-1.11; -0.33	0.06
medication intolerance	18	0.11	-0.41; 0.64	
spironolactone at baseline = yes	19	-0.77	-1.62; 0.07	0.61
spironolactone at baseline = no	51	-0.52	-0.91; -0.13	
Systolic office BP <188mmHg	35	-0.29	-0.74; 0.16	0.16
Systolic office BP ≥188mmHg	35	-0.72	-1.22; -0.23	
Isolated systolic hypertension = yes	13	-0.44	-0.93; -0.05	0.81
Isolated systolic hypertension = no	57	-0.58	-0.96; -0.19	
BMI <28.5kg/m	34	-0.22	-0.71; 0.26	0.15
BMI ≥28.5kg/m	36	-0.73	-1.20; -0.27	
Baseline eGFR <82ml/min/1.73m	35	-0.74	-1.23; -0.26	0.05
Baseline eGFR ≥82ml/min/1.73m	35	-0.15	-0.62; 0.32	
urinary sodium excretion <162mmol/24h	34	-0.49	-0.90; -0.08	0.72
urinary sodium excretion ≥162mmol/24h	36	-0.6	-1.11; -0.09	
smoking = no	60	-0.64	-0.98; -0.29	0.09
smoking = yes	10	0.29	-1.03; 1.61	

BP = blood pressure, BMI = body mass index, eGFR = estimated glomerular filtration rate. Estimated slope represents the estimated change in home blood pressure per month.

**Fig 2:**
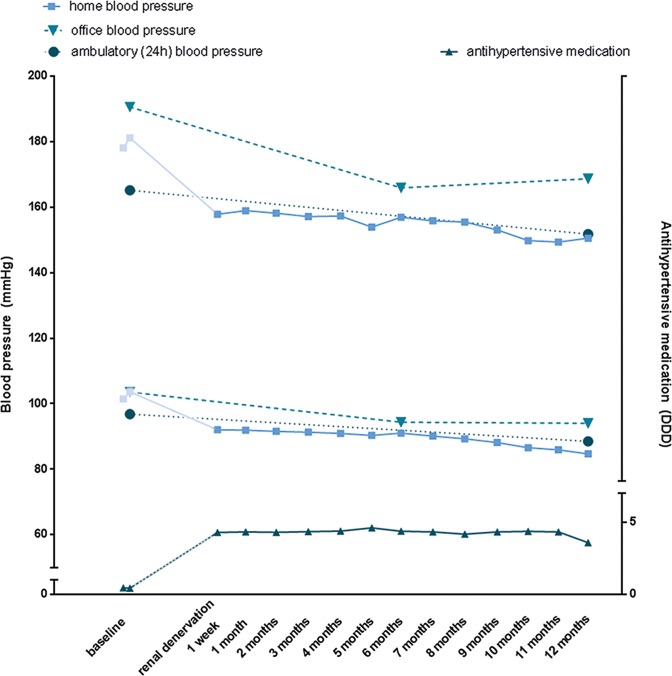
Changes in blood pressure measured by various modalities and antihypertensive medication over time. Figure depicts the change in blood pressure measured by various modalities (displayed on left axis) and antihypertensive medication (displayed on right axis). SD bars for BP overlapped for both baseline and 12-month follow-up, indicating lack of statistical significance, but were omitted in the figure for clarity.

Fig 2 shows the change of SBP measured by various modalities. Office BP decreased from 191/104 mmHg (SD 31/16) at baseline to 169/94 mmHg (SD 28/15) at twelve months follow-up, while mean 24-hour BP decreased from 165/97 mmHg (SD 16/16) to 152/89 mmHg (SD 22/14), levelling up with HBPM levels at 12 months follow-up. The rates of change for the various modalities are presented in Table 4.

**Table 4:** Rate of change in blood pressure measured by various modalities.

	HBPM		ABPM		OBPM	
	delta	95% CI	delta	95% CI	delta	95% CI
systolic	-8,1	(-12,0 to -4,2)	-9,3	(-14,2 to -4,4)	-15,9	(-23,8 to -7,9)
diastolic	-3,8	(-6,1 to -1,6)	-5,5	(-8,0 to -3,0)	-6,6	(-10,1 to -3,0)

Rate of change calculated as absolute reduction in mmHg per year. The reduction in HBPM was estimated using LMM. HBPM = home blood pressure measurements, ABPM = ambulatory blood pressure measurements, OBPM = office blood pressure measurements.

## Discussion

Our results show a number of important observations. First, a gradual decrease in BP following RDN was observed over the course of 12 months. Second, the observed decrease did not reach an obvious plateau during our follow-up and was unaffected by changes in the amount of antihypertensive medication. Lastly, the rate of change in home BP measurements and 24-hr ambulatory BP measurements was comparable, while office measurements appeared to be higher throughout the study.

These results provide an answer in the debate concerning the timing of the effect of RDN. It has been stated by some that RDN has a rapid effect during the first trimester, while others have advocated that the effect may take several months to occur.[25,26] In previous studies, the time intervals between BP measurements were too large to investigate the exact timing of effects. We performed monthly home BP measurements (HBPM), enabling us to investigate the dynamics of BP more accurately and to determine the timing of BP changes after RDN. Our results show that BP decreased in a gradual fashion without an obvious dip. This may indicate that BP changes after RDN indeed do not occur as an acute drop but simply as a gradual change over a long period of time, which is in line with previous studies that demonstrated further BP reductions at a similar rate at 24 and 36 months compared to 1 year follow-up.[27,28] That concept is further supported by the results of the DENERHTN trial[8] demonstrating a gradual decrease on HBPM throughout 6 months quite similar to ours, although the absolute decrease in HBPM was higher. The latter may be explained by the uptitration of antihypertensive medication based on the HBPM readings in the DENERHTN trial. The observed gradual decrease in both studies may also be due to considerable interpersonal variety in the occurrence of BP effects that are levelled off at group level. The observations would imply that BP management can benefit greatly from implementing HBPM in the routine follow-up after RDN. Frequent HBPM allows for quick detection of BP changes and subsequent adjustment of antihypertensive treatment that may be delayed in the case of occasional office or ambulatory measurements.

An interesting finding in our study is that, in contrast to patients with resistant hypertension, patients with medication intolerance did not show a decrease (if anything, a slight increase) in blood pressure. This is in contrast to the findings of De Jager et al, who demonstrated significant decreases in office and ambulatory blood pressure in a small cohort of patients not taking AHD.[29] Obtaining measurements unbiased by antihypertensive medication is important, since changes in adherence may influence the results after RDN in opposing ways: decreased adherence may mask the effects of renal denervation, while increased adherence may induce an apparent RDN effect that is not really there. Therefore, further research involving a not treated hypertensive population will be of special interest.[30]

HBPM also has additional value in hypertension research. Previous studies in the field of RDN have mostly used office BP measurements as an endpoint. However, it is known that office BP measurements are subjected to several disadvantages, such as observer bias and white coat effect. Even when performed under ideal circumstances, such as proper positioning of the patient, well-trained personnel and selection of the correct cuff size, office BP readings have poor reliability and tend to overestimate true BP.[31–33] This is also reflected by the higher baseline and follow-up values for office BP in our analysis. ABPM and HBPM provide better reproducibility, are more accurately related to the real BP of daily life and are not subjected to the white coat effect.[19] In turn, HBPM has the advantage over ABPM that it is cheaper, more convenient for the patient and allows multiple measurements over longer periods of time.[34,35]

Randomised sham-controlled trials, such as the HTN-3 trial[10] and the study by Desch et al.,[11] are of superior methodology to assess therapeutic efficacy and have failed to demonstrate a statistically significant benefit of RDN compared to a sham procedure. Still, these studies have investigated the patient under highly controlled circumstances, whereas our patients were studied in a setting that reflects real life.

### Strengths and Limitations

We studied blood pressure changes after RDN using HBPM, which is considered more reliable than office measurements and has not yet been widely used in RDN research. Using HBPM also minimized the influence of regression-to-the-mean in our analysis, because the effect of this statistical phenomenon quickly diminishes after a few readings[36] and HBPM uses the mean of many BP measurements. Furthermore, HBPM also allowed us to investigate BP changes in a real life setting, as opposed to the highly controlled hospital setting of clinical trials.

As stated in the introduction, recent trials raised several issues concerning the effectiveness of RDN, including technical aspects, anatomical issues, patient selection, study design and timing of the BP response. In the current analysis we were able to address the latter, but other aspects remained unaddressed. As long as a quantitative measure for the extent of nerve damage effectuated by the RDN procedure is lacking, any statements concerning causality are highly speculative. Therefore, we can make a statement regarding *when* BP reduction occurs after RDN, but we cannot provide an answer in the discussion whether the observed effect is *caused* by the intervention. Although we did observe an apparent drop in BP between baseline and the first measurement after RDN (one week post-RDN), this observation is biased by an artificial increase in antihypertensive medication caused by the medication free screening period and therefore not included in the LMM analysis. Therefore, we can neither rule out nor demonstrate the coexistence of an acute drop in BP during the first days.

Lastly, we did not include a control group and therefore cannot compare the BP effect in our intervention group to BP control measured by HBPM in a hypertensive population without intervention. The observed BP effect in our study was modest and the use of HBPM may have contributed to the BP reduction as it may not only be used as a diagnostic, but also as an educational tool. Especially when combined with telemonitoring, HBPM can contribute to better BP control and the need for less antihypertensive medication.[37–39] However, this effect, if present, is likely to be small: in a meta-analysis of 37 studies with a duration up to 36 months, the average effect of HBPM and telemonitoring on BP was less than 3 mmHg systolic.[40]

### Conclusions

In this study we have evaluated BP dynamics the first year after RDN. Using frequent home BP monitoring in a real life setting we demonstrated a gradual decrease over time after RDN. Future research needs to distinguish whether this decrease represents a true effect of RDN, or whether it is effectuated through other factors as discussed above. Particularly in hypertension research, the use of a randomized sham-controlled design and reliable BP measurements is key. For outcome measurement in these studies, HBPM may be a more informative and convenient alternative to ABPM. However, it is important to realize that any statements concerning causality between the RDN procedure and the observed effect on BP are highly speculative as long as a quantitative measure for the extent of nerve damage is lacking.
